# An In-Situ Electrochemical Nanoindentation (ECNI) Study on the Effect of Hydrogen on the Mechanical Properties of 316L Austenitic Stainless Steel

**DOI:** 10.3390/ma14216426

**Published:** 2021-10-26

**Authors:** Adina Basa, Dong Wang, Nuria Espallargas, Di Wan

**Affiliations:** Department of Mechanical and Industrial Engineering (MTP), Norwegian University of Science and Technology (NTNU), Richard Birkelands vei 2B, 7491 Trondheim, Norway; adina.basa@ntnu.no (A.B.); dong.wang@ntnu.no (D.W.); nuria.espallargas@ntnu.no (N.E.)

**Keywords:** hydrogen embrittlement, in-situ electrochemical nanoindentation, austenitic stainless steel, electron backscattered diffraction, cathodic charging

## Abstract

In-situ electrochemical nanoindentation (ECNI) has been used to study the effect of hydrogen on the mechanical properties of austenitic stainless steel AISI 316L. Changing the electrode potential (via electrochemical charging) revealed the interconnected nature of the hydrogen effect on the nanomechanical properties of the stainless steel. At more positive cathodic potentials, a softening effect of hydrogen can be noticed, while significant hardening can be observed at more negative cathodic potentials. The hydrogen effects on the nanomechanical properties were analyzed in terms of the homogeneous dislocation nucleation (HDN) and the hydrogen-dislocation interactions from the energy point of view. The effects can be explained with the framework of the defactant theory and the hydrogen-enhanced localized plasticity (HELP) mechanism.

## 1. Introduction

The interaction between hydrogen and structural materials, especially stainless steels, is causing numerous kinds of problems in industry, among which hydrogen embrittlement has been a great concern since its first discovery in 1875 [1]. In many industrial applications, the ingress of hydrogen into the steel microstructure has effects on the chemical and mechanical properties of the materials. There are different mechanisms in the literature explaining the hydrogen-induced degradation, such as hydrogen-enhanced localized plasticity (HELP) [2,3,4], hydrogen-enhanced decohesion (HEDE) [5,6,7], hydrogen-enhanced strain-induced vacancy formation (HESIV) [8,9], adsorption-induced dislocation emission (AIDE) [10,11], etc. More recently, researchers found that the mechanisms are not independent and can sometimes work simultaneously to have a synergistic action. For instance, the HELP + HEDE concept or the HELP-mediated HEDE model has been proposed [12]. However, a common agreement on what are the main causes for hydrogen embrittlement is still challenging to find due to the intrinsic complexity of the testing procedures and the many factors acting simultaneously in the process. This can in fact be seen as a system-dependent process, in which the operating conditions (environment, electrolyte composition, mechanical action, etc.) will determine the response, and therefore it is very difficult to have a unique theory or explanation for this degradation mechanism.

Austenitic stainless steels (ASS) are widely used in a variety of applications where corrosion resistance is needed, like in the oil and gas industry, offshore structures, or in pressurized pipeline applications [13,14,15]. In these applications, the risk of hydrogen embrittlement exists since the system is typically exposed to hydrogen (via cathodic protection) and mechanical loading [16,17,18]. The mechanical properties of the stainless steels exposed to cathodic charging or hydrogen gas environments degrade very quickly since hydrogen is a small atom that can very easily diffuse in the microstructure of metals [19,20,21,22]. There is a vast number of experimental studies focusing on the relationship between hydrogen and plastic deformation in ASS, reporting significant effects on the plasticity [23,24,25,26]. However, a thorough literature survey shows a large contradiction in the magnitude of the hydrogen-induced degradation of mechanical properties. To justify the apparent inconsistency of some of these results, the experimental conditions (e.g., hydrogen charging conditions, geometry of the sample, stability of the structure) must be carefully controlled. For example, hydrogen charging of ASS at high temperatures may provide a relatively uniform distribution of hydrogen due to the higher diffusion coefficient than at room temperature [27,28]. Another example is the ex-situ mechanical tests, which are affected by the hydrogen gradient in the sample as a result of the outgassing of the hydrogen throughout the tests. The in-situ electrochemical hydrogen charging at room temperature can also be affected by the hydrogen gradient from the surface into the bulk of the sample [29]. In addition, the in-situ electrochemical hydrogen charging using recombination poisons like As_2_O_3_ (to increase the hydrogen effects) may lead to lattice distortions of the austenite as well as an increase of the defect density and the local formation of unstable hydrides [30,31]. Even though these observations report the degradation of mechanical properties due to hydrogen, they cannot be accounted for in the interaction of hydrogen with dislocations during plastic deformation.

The electrochemical nanoindentation (ECNI) technique has been proven to be a reliable approach to study the effect of hydrogen on materials’ plasticity due to a uniform hydrogen concentration on the sample surface as well as the fact that the sample surface quality is not altered during hydrogen charging. ECNI implies using moderate conditions, i.e., low current densities are applied and no additional recombination of poisons are required, and therefore the austenite stability is not affected [32]. ECNI brings the possibility of simultaneous electrochemical hydrogen charging and nanomechanical testing within a very small volume close to the surface where the hydrogen concentration within a very short time becomes uniform. The significant advantage of ECNI is that all related behavior can be limited within a single grain, such that microstructural variations can be eliminated as much as possible. Moreover, when the microstructural features are intentionally studied, by combining micro-fabrication techniques, the small-scale cantilever beam bending or pillar compression tests can provide more insights [33,34,35,36]. Since ECNI is focusing on the deformation behavior in a limited volume, getting comparable results as from large-scale testing is rather challenging, but on the other hand, it suits refined studies on the genuine local material behavior. This technique has already been applied for studying the mechanism of hydrogen embrittlement and hydrogen dislocation interaction in various materials [37,38,39,40]. To the authors’ knowledge, ECNI studies on ASS are relatively limited [41,42]. While previous works mostly focused on the hardening effect of hydrogen with cathodic polarization, sometimes, it can also be found that a softening effect can be recorded, similar to that from large-scale tests [12,43]. Covering a wider polarizing potential range (therefore a variety of hydrogen amounts) in the studies may potentially provide information for better understanding the hydrogen effect in a more comprehensive way.

In this paper, in-situ ECNI was used to study the effect of hydrogen on the nanomechanical performance of austenitic stainless steel AISI 316L. During the nanoindentation of samples with a very low dislocation density, it is possible to observe a homogeneous dislocation nucleation (HDN) below the surface. By further analyzing the pop-in behavior, the energy consumption can be carefully modelled during the onset of plastic deformation. By controlling the electrochemical cathodic potentials, different amounts of hydrogen can be introduced, and thus the nanomechanical performance evolution can be clearly observed with respect to hydrogen evolution. Finally, the possible hydrogen–metal interaction mechanisms are discussed. The current methodology can avoid extrinsic influencing factors as much as possible and reveal the intrinsic hydrogen–material interaction.

## 2. Materials and Methods

### 2.1. Sample Preparation

A cylindrical sample (12 mm diameter and 1~2 mm thickness) was cut by electrical discharge machining from a 316L ASS slab. The composition is given in Table 1. In order to achieve large austenite grains in the microstructure (ca 200 µm) and a low dislocation density, the sample was heat treated at 1150 °C for 8 days in vacuum followed by cooling in the furnace.

Standard surface preparation, including grinding with silicon-carbide papers up to 2400 grade, followed by mechanical polishing with a water-based diamond suspension up to 1 µm was carried out. In the final step, electropolishing using 20 V in a methanol/H_2_SO_4_ electrolyte for 30 s was used. Electropolishing was used to remove the plastically deformed surface layer produced by mechanical polishing. This is an important step in studying the metastable ASS because the mechanically deformed layer can undergo a phase transformation (austenite to martensite) and the electropolishing step guarantees a fully austenitic sample.

### 2.2. Microstructure

**Figure 1:**
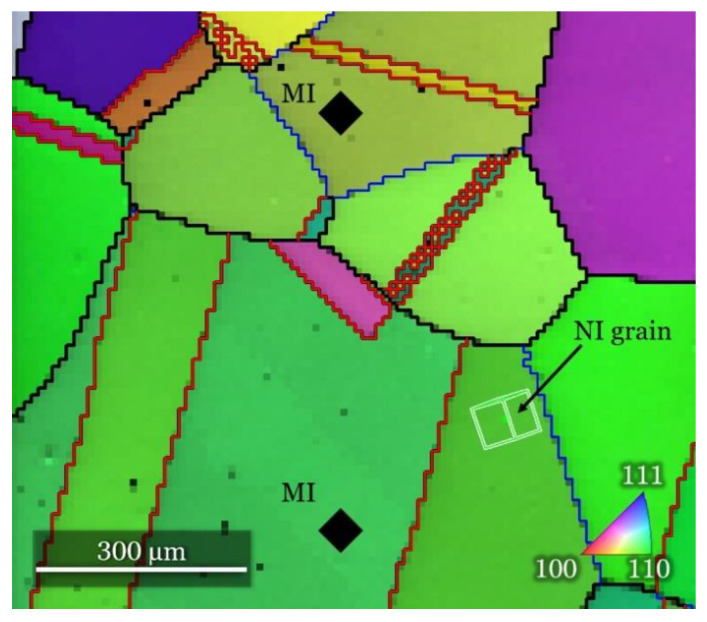
Normal direction inverse pole figure map of 316L ASS sample marked with microindents (MI) for finding the grain selected for performing nanoindentation test. Black line is high-angle grain boundary; blue line is low-angle grain boundary; red line is twin boundary with 60°-<111> relationship.

The microstructure of the material was characterized by electron backscatter diffraction (EBSD) mapping. A field emission scanning electron microscope (FESEM) Zeiss Supra 55 VP (ZEISS, Jena, Germany) was used for EBSD mapping of the samples prior to testing and TSL OIM software (version 7.2.1) was used for the data analysis. The sample was tilted by 70°, the acceleration voltage was 30 kV, the working distance was 21 mm, and a step size of 200 nm was used for EBSD mapping. Figure 1 presents the normal direction (i.e., indentation direction) inverse pole figure map. The initial microstructure of the tested material consisted of well-annealed equiaxial grains with a grain size larger than 200 µm. To exclude the crystallographic orientation effect, all sets of nanoindentations were performed within the same grain oriented close to the (101) direction, as indicated by the arrow in the figure. For easier identification of the selected grain, the sample was marked with 2 microindents (denoted as MI in Figure 1) where the prints of the microindents are sufficiently deep to be visible with the optical microscope of the TI-950 nanoindentation system (Bruker, former Hysitron, Minneapolis, MN, USA). Notice that the distance between the microindents and the grains is sufficiently large (larger than 200 µm), so the investigated grain is not affected by the plastic zones of the microindents.

### 2.3. In-Situ ECNI

**Figure 2:**
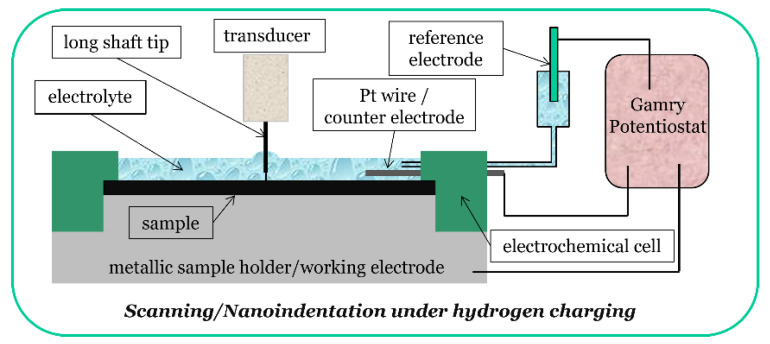
Experimental setup of the in-situ ECNI technique.

The in-situ ECNI experiments were performed with a Tribo-Indenter TI-950, with a Performech^TM^ controller (Bruker, former Hysitron, Minneapolis, MN, USA) in combination with an electrochemical setup to allow hydrogen charging of the samples. In addition to nanoindentation, the TI-950 is capable of scanning prior to and after testing, giving topographical images of the samples. A long shaft Berkovich indenter tip was calibrated for both the tip area function and machine compliance prior to the tests. The schematic of the experimental setup is shown in Figure 2.

**Figure 3:**
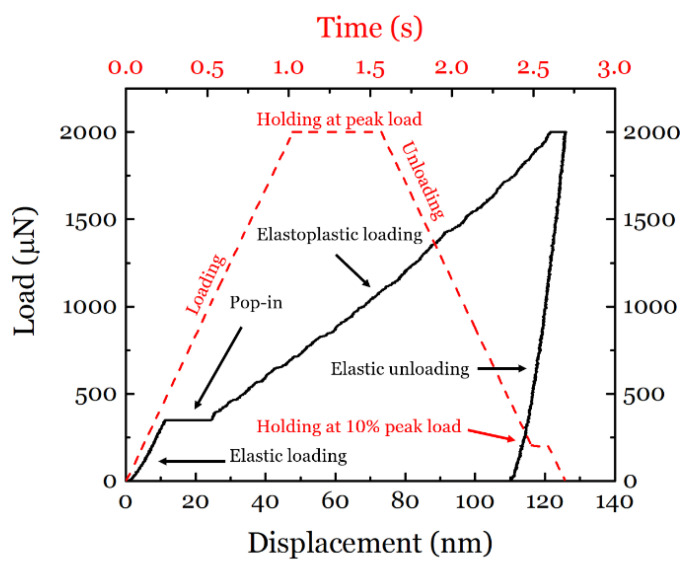
Nanoindentation load function (red) and a typical resulted load–displacement curve (black).

The nanoindentation was performed with a maximum load of 2000 µN and a loading rate of 2000 µN/s. A typical load–displacement curve together with the load function is presented in Figure 3. For drift corrections, an additional holding time of 0.2 s at 10% of the maximum load value was added during the unloading. For hydrogen charging, a three-electrode electrochemical setup was used. The electrolyte was prepared from Na_2_SO_4_ (99%, Merck KGaA, Darmstadt, Germany) with deionized water at a concentration of 0.05 mol/L. The measured pH was 6.22. A platinum wire was used as the counter electrode, while Hg/Hg_2_SO_4_ was used as reference electrode. All electrode potentials in this study are referenced to this electrode. The working electrode was the ASS sample, and a Gamry Reference 600^TM^ Potentiostat (Gamry Instruments Inc., Warminster, PA, USA) was used to control the electrode potentials. Scanning probe microscopy (SPM) and nanoindentation tests were performed inside the electrochemical cell while the sample surface was immersed in the electrolyte.

**Figure 4:**
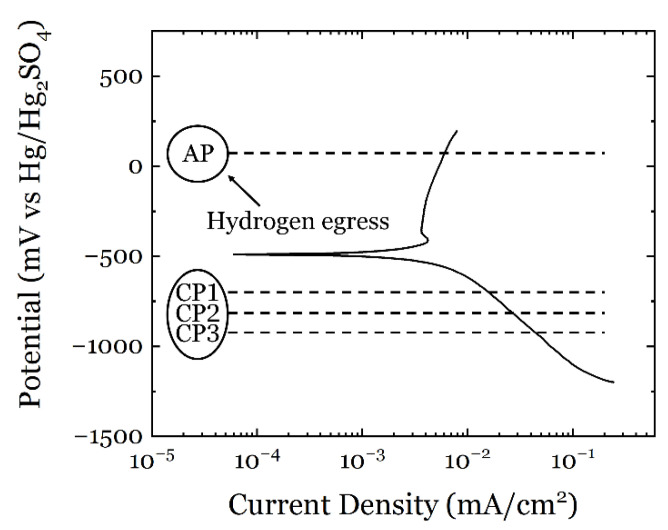
Polarization curve of the tested ASS at a scanning rate of 5 mV/s. AP is the anodic potential of +100 mV, CP1 is the cathodic potential of −740 mV, CP2 is the cathodic potential of −808 mV and CP3 is the cathodic potential of −884 mV.

Before the ECNI test, the polarization curve of the ASS sample was measured at a scanning rate of 5 mV/s using the same setup as in the ECNI test, and the results are shown in Figure 4. The polarization curve was repeated at least three times, but only one plot is shown in the figure for clarity.

The polarization curve shows the typical passive behavior of an ASS in a slightly acidic solution. The polarization curve shows three potential domains, i.e., cathodic, E_corr_, and anodic. The cathodic domain comprises the potential range below −500 mV (corresponding to the corrosion potential, E_corr_), where the current is determined by the reduction of dissolved oxygen as it corresponds to a slightly acidic medium (pH of 6.22). The second potential domain is characterized by the transition from cathodic to anodic current at the corrosion potential, E_corr_ (−500 mV). At E_corr_, the speed of the anodic and cathodic reaction is equal. The third domain corresponds to the anodic region, where oxidation of the metal takes place. In this case, this is a passive metal and two phenomena are observed in the anodic region: (1) active dissolution of the metal (−500 to −400 mV) characterized by an increase in current, and (2) passivation of the metal starting at −400 mV, which is characterized by a constant current density due to the passive film formation (a very thin, between 2 and 10 nm, homogeneous and uniform oxide layer mostly composed of chromium oxides builds up). At the tested conditions, no transpassive region (dissolution of the passive film and other substances) is visible in the polarization curve.

The electrode potentials chosen for the ECNI tests are denoted in Figure 4 with AP, CP1, CP2, and CP3. The testing sequence is presented in Table 2. The AP is the anodic potential located in the passive region of the ASS, and at this condition a nanometric thin oxide film (mostly chromium oxide) is present on the surface of the metal. The cathodic potentials (CP1 to CP3) are located in different regions of the cathodic domain where different reactions take place. The electrolyte chosen for this study is an aerated aqueous solution with a slightly acidic pH. Therefore, the cathodic reactions expected are the following:

O_2_ + 4H^+^ + 4e^−^ → 2H_2_O (in the potential range −500 mV~−740 mV) (R1)

H^+^ + 2e^−^ → H_2_ (in the potential range below −740 mV) (R2)

At CP1 (−740 mV), no or very little hydrogen evolution is expected in the current testing conditions since this value corresponds to the redox potential of hydrogen at pH 6.22 [44]. In the potential range between E_corr_ and CP1, oxygen is present in the solution and the metal surface will be passive even though a cathodic potential is being applied. In this potential range, only reaction 1 takes place. At CP2 and CP3, both reduction reactions (1 and 2) take place simultaneously and hydrogen gas starts to form on the surface of the stainless steel, promoting depassivation of the metal surface (i.e., a very thin passive film is still expected, thinner than for AP and CP1). The reduction reaction 2 exclusively takes place at electrode potentials below −1200 mV where the slope of the cathodic curve changes. At electrode potentials below −1200 mV no passive film is expected on the metal surface and only hydrogen evolution will take place.

The testing conditions are shown in Table 2. The testing procedure (including nanoindentation) was as follows: first, a freshly electropolished sample was mounted in the electrochemical cell and tested in air conditions. Then, the cathodic potentials were applied while the electrolyte was added to the sample. The nanoindentation tests were performed in different locations of the same grain after cathodic polarization (CP) by the time indicated in Table 2. The polarization plus testing sequence was repeated until all the electrode potentials were tested. The last step was done at the anodic potential (AP) indicated in Figure 4 and Table 2. Note that the AP time was intentionally designed longer than CPs to egress the hydrogen as much as possible. After the nanoindentation testing of each polarization sequence, the topography of the sample surface was inspected by SPM.

### 2.4. Statistical Analysis

**Figure 5:**
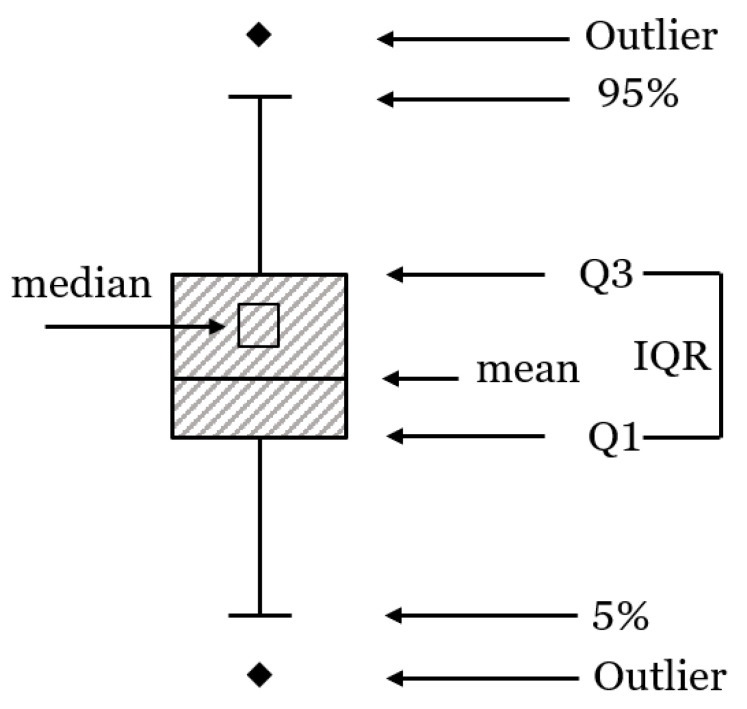
Interpretation of the box plot.

The statistics collected from the nanoindentation tests are presented using a box plot (or box-whisker plot) in the following sections for better visibility of the data and the associated statistical distribution. The interpretation of the plot is shown in Figure 5. The major shaded box symbol in Figure 5 shows the data range by their first (Q1) and third (Q3) quartiles (i.e., 25% and 75%), which is defined as the interquartile range (IQR). In the shaded box, the median value and the mean value are presented by a smaller box and a cross-line, respectively. The upper and lower whiskers show the 95th percentile and the 5th percentile, respectively, excluding any outliers. The outliers are also plotted as diamond symbols. Note that not all data sets have outliers. In the current study, at least 25 repetitions were performed for each nanoindentation testing condition.

## 3. Results

### 3.1. Load-Displacement Curves

**Figure 6:**
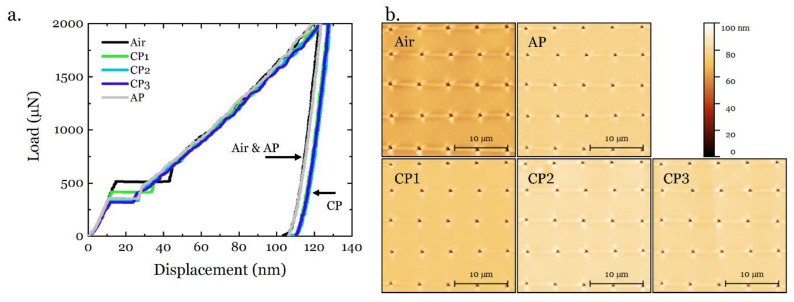
(**a**). Representative LD curves for different charging conditions and (**b**). the corresponding SPM images. Note the color scale applies to all SPM images.

The sequence of the testing conditions applied to the sample are given in Table 2 and the representative load–displacement (LD) curves resulting from the nanoindentations under different potentials are shown in Figure 6a. All the LD curves clearly show four stages, namely elastic loading, pop-in, elastoplastic loading, and the final elastic unloading. When measured in air and under AP, the LD curves are very similar, while there is a clear change in the LD curves under CP. Note that the CP conditions were sequentially applied after the air condition and before the AP condition. Therefore, the LD behavior experienced a detectable change under CP and recovered to that under the initial air condition. Based on the LD results, special attention is paid to the variations (including hardness, reduced modulus, pop-in load, and pop-in width) at different electrochemical potentials, and a comprehensive analysis of the behavior of the selected steel is performed. The surface roughness was scanned after each set of tests and the scanning results are shown in Figure 6b. The SPM images do not reveal significant roughness on the surface after the individual polarization, and thus it can be concluded that the changes in the material behavior were due to the dissolved hydrogen and not to the changes in the surface conditions. Furthermore, the holding stage at 10% peak load in the unloading segment (see Figure 3) did not reveal significant changes in the LD curves for all conditions. Therefore, the thermal drift can be neglected in the ECNI procedure. The post-analysis results from the LD curves are presented in the following sections.

### 3.2. Hardness and Elastic Moduli

A deeper analysis of the hydrogen effect on mechanical properties is performed and the LD curves were used to extract hardness and elastic modulus according to the Oliver–Pharr method. The resulting hardness (*H*) values and effective Young’s moduli (reduced moduli *E_r_*) can be calculated from Equations (1) and (2) [45]:

(1) $$H=\frac{P_{max}}{A_{c}}$$

(2) $$E_{r}=\frac{S}{2\beta }\sqrt{\frac{\pi }{A_{c}}}$$

where *P_max_* is the maximum applied load and *A*_c_ is the tip area function that represents the projected area at a given contact depth *h_c_*, *S* is the material stiffness, and *β* is a correction factor depending on the tip geometry (1.034 for a Berkovich indenter) as in Equation (3):

(3) $$h_{c}≅h_{max}-0.75\frac{P_{m}}{S}$$

In Equation (3), *h_max_* represents the maximum displacement reached by the tip and the stiffness *S* is extracted from the initial unloading slope of the LD curves.

**Figure 7:**
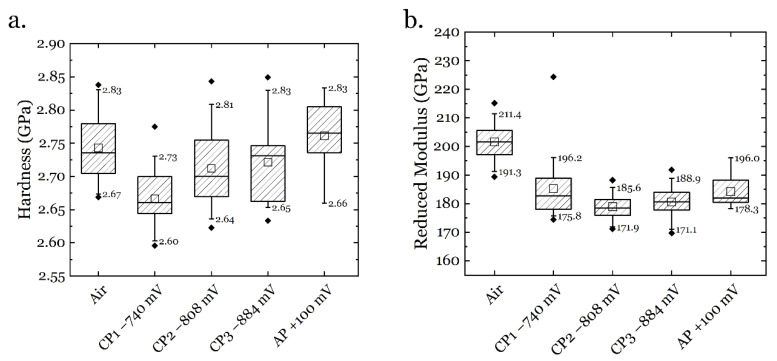
(**a**). Hardness and (**b**). reduced modulus under different charging conditions.

By combining the above equations, the hardness and reduced modulus can be calculated from the LD curves, and the results are presented in Figure 7. Without any electrochemical treatment in air, the specimen showed a mean hardness of 2.74 GPa. When CP1 was applied, the hardness slightly decreased to 2.67 GPa (about 2.5%). By further increasing the polarization potential to CP2 and CP3, the hardness recovered to 2.71 GPa and 2.72 GPa, respectively. By applying AP at +100 mV, the hardness fully recovered to 2.76 GPa, which is slightly higher than that in the air condition. On the other hand, the reduced modulus has a slightly different trend. The initial reduced modulus was 201.6 GPa in the air condition. When applying hydrogen charging at CP1~3, it decreased to 185.3 GPa, 179.0 GPa, and 180.1 GPa, respectively. After the egression of hydrogen under AP, the reduced modulus increased to 184.2 GPa, which is still lower than the air case.

### 3.3. Pop-In Behavior

**Figure 8:**
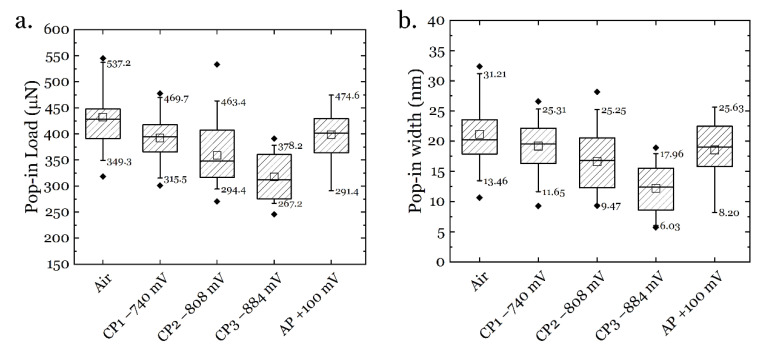
Pop-in data from the ECNI under different charging conditions: (**a**). pop-in load; (**b**). pop-in width.

The pop-in phenomenon captured by the nanoindentation technique is believed to be triggered by the onset of plastic deformation [46], and upon in-situ hydrogen charging, a lowered pop-in load is always observed [47,48]. The pop-in data from the ECNI tests in the current study are summarized in Figure 8. The pop-in procedure can be depicted by the energy-based model proposed by Wang et al. [40].

Assuming the onset of plastic deformation results from the elastic stored energy (i.e., the loading segment before the pop-in starts), based on the energy balance principle that the elastic stored energy is consumed by the formation of dislocations with the associated interaction and lattice friction, the following mathematical relationship in Equation (4) can be assumed:

(4) $$W_{e}=W_{i}^{tot}+N\cdot W_{s}+W_{f}^{tot}$$

where *W_e_* is the elastic stored energy, $W_{i}^{tot}$ is the total interaction energy between dislocation, *N* is the number of generated dislocation loops, *W_s_* is the dislocation line energy, and $W_{f}^{tot}$ is the total friction energy on dislocation motion during pop-in.

Since the elastic loading part can be described by the Hertzian contact theory, the stored energy can be described by Equation (5):

(5) $$W_{e}=\int_{0}^{h_{1}}P\left(h\right)dh=\int_{0}^{h_{1}}\frac{4}{3}E_{r}h^{\frac{3}{2}}R^{\frac{1}{2}}dh=\frac{8}{15}E_{r}R^{\frac{1}{2}}h_{1}^{\frac{5}{2}}$$

where *P* and *h* denote the load and depth during indentation, and *h*_1_ means the start depth of the pop-in. *R* is the tip radius that equals to 1875 nm in the current study.

The interaction energy between two circular prismatic dislocation loops can be described as Equation (6):

(6) $$W_{i}=\frac{\mu b^{2}}{1-\nu }r\left(\ln\frac{8r}{d}-1\right)$$

where *µ* stands for the shear modulus, *b* denotes the magnitude of the Burgers vector, *ν* the Poisson’s ratio, and *d* the distance between dislocation loops which is assumed to be equal to *b*, considering dislocation loops are piled on the close-packed crystallographic plane. The radius of dislocation loops *r* is determined from the stress field beneath the indenter by assuming the 98% maximum shear stress, as proposed in [40]. Therefore, the radius *r* can be correlated with the contact depth *a_c_* in Equation (7):

(7) $$r=0.29a_{c}=0.29\times \sqrt[{3}]{\frac{3PR}{4E_{r}}}$$

The total interaction energy is calculated by considering the interactions between each dislocation loop with all the rest loops, which gives the following relationship in Equation (8):

(8) $$W_{i}^{tot}=\frac{\mu b^{2}}{1-\nu }r\left(\left\{\overset{N-1}{\underset{j=1}{\sum }}j\left(\ln\left(\frac{8r}{d}\right)\right)-\overset{N-1}{\underset{j=1}{\sum }}\ln\left(j!\right)-\overset{N-1}{\underset{j=1}{\sum }}j\right\}\right)$$

The number of generated dislocation loops can be correlated with the pop-in width Δ*h* in Equation (9):

(9) $$N=\frac{\Delta h}{2b}$$

The dislocation line energy is expressed by Equation (10):

(10) $$W_{s}=\frac{\mu b^{2}}{2\left(1-\nu \right)}r\left(\ln\frac{8r}{\rho }-1\right)$$

Here, *ρ* is the radius of dislocation core which is assumed to be equal to *b*/2.

By combining Equations (4)–(10), the total friction energy $W_{f}^{tot}$ for dislocation motion during pop-in can be calculated. The unit friction energy (*W_f_*, i.e., the energy consumed for a single dislocation loop to move a distance of *b*) can be correlated with the total friction energy by Equation (11):

(11) $$W_{f}\cdot \frac{N\left(N-1\right)}{2}=W_{f}^{tot}$$

**Figure 9:**
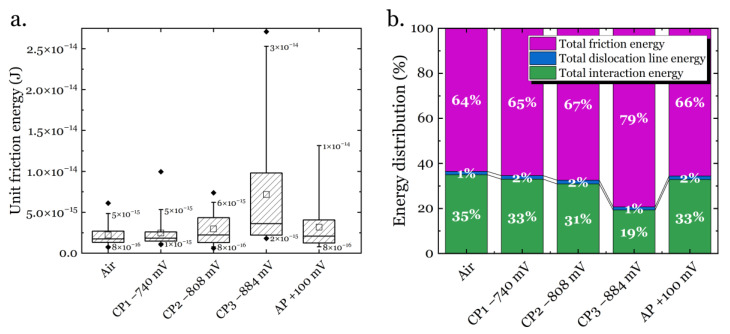
Lattice friction analysis based on the pop-in behavior from ECNI under different charging conditions: (**a**) unit friction energy; (**b**) energy consumption distribution during pop-in.

Finally, the unit friction energy for individual dislocation motion and the corresponding energy consumption distribution for the entire pop-in procedure can be evaluated. The results are presented in Figure 9. Although the calculated friction energy with the scatter range looks similar between different charging conditions, a consistent trend can still be depicted regarding the hydrogen ingression and egression (see Figure 9a). In the air condition, the unit friction energy was calculated to be 1.84 × 10^−15^ J, and after charging at CP1 to CP3, the unit friction energy increased to 2.05 × 10^−15^ J, 2.28 × 10^−15^ J and 4.34 × 10^−15^ J, respectively. After the hydrogen egression by AP, the unit friction energy recovered to the level of 2.09 × 10^−15^ J. When considering the energy consumption categories (see Figure 9b), the friction occupied 64% of the total energy during pop-in, and this value increased to 65%, 67%, and 79% under CP1, CP2, and CP3, respectively. After hydrogen egression, this portion dropped again to 66%. The competition was mainly between the friction and the dislocation interactions in the energy consumption, and in comparison, the energies consumed by dislocation line formation did not vary noticeably and engaged about 1~2% for all the testing conditions.

### 3.4. Homogeneous Dislocation Nucleation (HDN)

During nanoindentation, the contact between the tip and the sample surface is under elastic loading until the first dislocation nucleation occurs, which represents the beginning of plasticity, marked by the pop-in in Figure 3. The microstructure of well-annealed equiaxial grains and proper electropolishing indicate a relatively low dislocation density of the tested specimen (usually about 10^10^ to 10^14^ m^−2^, corresponding to an average dislocation spacing of approximately 1–10 µm [39], which is much larger than the indentation depth). Thus, it is reasonable to consider that at the onset of the pop-in during nanoindentation, the maximum shear stress under the indenter, *τ_max_*, can be the shear stress resulting in HDN. According to continuum mechanics [49,50], the value of the maximum shear stress *τ_max_* that appears at the position *z_τ_*_(*max*)_ is given by:

(12) $$\tau_{max}=0.31{\left(\frac{6E_{r}^{2}}{\pi^{3}R^{2}}P\right)}^{\frac{1}{3}}$$

(13) $$z_{\tau \left(max\right)}=0.48{\left(\frac{3PR}{4E_{r}}\right)}^{\frac{1}{3}}$$

where *E_r_* is the reduced modulus, *R* is the tip radius, and *P* is the pop-in load. The tip radius is extracted from fitting a Hertzian model to the elastic loading part of the LD curves, which is 1875 nm for the current study. Thus, the maximum shear stress obtained from Equation (12) is responsible for the HDN at *z_τ_*_(*max*)_ below the tip.

According to classic dislocation theory, the formation of a circular dislocation loop with radius *r* requires a free energy given by [49]:

(14) $$\Delta G=2\pi r\gamma_{dis}-\pi r^{2}b\tau_{max}+\pi r^{2}\gamma$$

The first term in the equation, $2\pi r\gamma_{dis}$ represent the line energy of the dislocation loop, the second term, $\pi r^{2}b\tau_{max}$ describe the work needed for expanding the dislocation loop, while the latest one is the formation of the stacking fault energy (SFE). Thus, $\gamma_{dis}$ (Equation (15)) represents the elastic self-energy of a full circular dislocation loop in an infinite isotropic elastic solid, γ is the SFE in mJ/m^2^ (which is 22.83 mJ/m^2^ according to Equation (16) [51]), and *b* is the Burgers vector for dislocation (0.254 nm [41]).

(15) $$\gamma_{dis}=\frac{2-\nu }{1-\nu }\frac{\mu b^{2}}{8\pi }\left(\ln\frac{4r}{\rho_{core}}-2\right)$$

(16) γ=2.2+1.9Ni−2.9Si+0.77Mo+0.5Mn+40C−0.016Cr−3.6N

where *µ* is the shear modulus (80 GPa for the 316L steel [41]), *ν* is Poisson’s ratio (0.3 [41]), and $\rho_{core}$ represents the dislocation core radius.

Hence, by using Equation (15), the free energy required for a dislocation loop formation can be expressed as:

(17) $$\Delta G=\frac{2-\mu }{1-\mu }\frac{Gb^{2}r}{4}\left(\ln\frac{4r}{\rho_{core}}-2\right)-\pi r^{2}b\tau_{max}+\pi r^{2}\gamma$$

**Figure 10:**
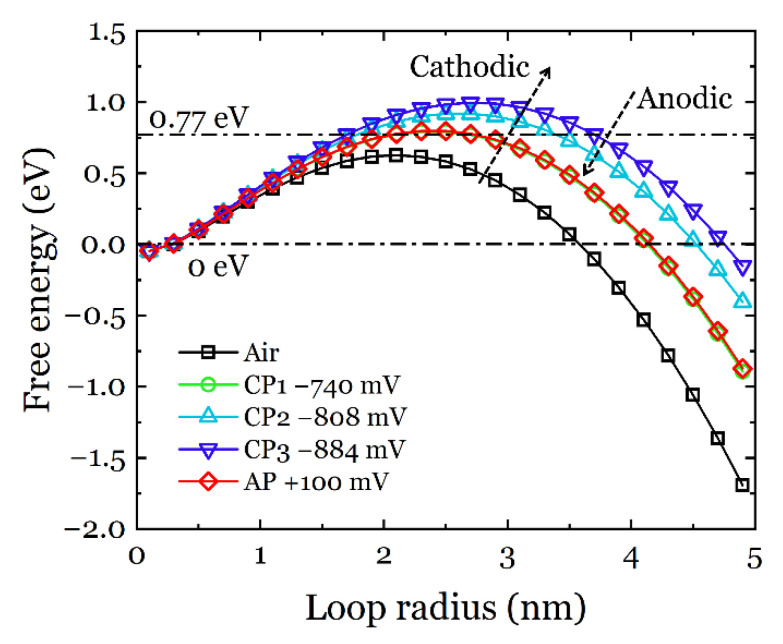
Free energy change of HDN as a function of dislocation loop radius calculated from the ECNI results.

The free energy change of HDN as a function of dislocation loop radius can be calculated by combining Equations (12)–(17), and the results are presented in Figure 10. The calculated curves have a maximum free energy ΔG* value at a critical loop radius r*. For the formation of a stable dislocation loop larger than r*, the activation energy has to pass the barrier of ΔG*. From Figure 10, considering the air condition as an initial reference, the ΔG* and the r* values are increasing with a higher cathodic potential, and after anodic discharging, the ΔG* and the r* values recovered towards the air condition, but not completely (the curve from AP is almost overlapping with the CP1 condition). This result is not surprising since at CP1 not enough hydrogen is formed. Indeed, since CP1 corresponds to the redox potential of hydrogen in the given testing conditions, one should expect a fully passivated surface in the material, very similar to the AP condition. In order to fully depassivate a stainless-steel surface, the cathodic potential applied should be below the redox potential for hydrogen. In the present study, this was found to be below −1200 mV (see Figure 4).

The constants used in the current study are summarized in Table 3.

## 4. Discussion

### 4.1. The Influence of Hydrogen on the Pop-In Behavior

The influence of hydrogen on the pop-in behavior of materials during in-situ ECNI has been a focus since the development of this technique, as the change in pop-in is always noticeable in the testing procedure. A general trend is that hydrogen reduces both pop-in load and pop-in width [39].

It has been proposed that the pop-in width can be related to the number, mobility, and interactions between dislocations during pop-in [52,53,54,55,56]. Recently, this procedure was modeled [40] using the energy balance criteria and the analysis for the current work has been presented in Section 3.3. From Figure 9a, the hydrogen evolution by CP has a clear enhancing effect on the lattice friction for dislocation motion, and this enhancement becomes greater when the potential increased in the cathodic direction. The accumulation of hydrogen atoms in the specimen could generate a Cottrell-like atmosphere that provides more resistance to the dislocation motion in the framework of the solute drag theory [57,58] (further discussion in Section 4.3). Therefore, more energy needs to be consumed to overcome the friction during the onset of plastic deformation. It is worth noting that the total energy consumption by friction quantitatively increases as the electrode potential increases in the cathodic direction (Figure 9b), which provides more evidence on the hydrogen-induced friction hypothesis. When the hydrogen was discharged by applying an anodic potential, the friction recovered towards the original level, though not completely. This irreversible property change could possibly be ascribed to three reasons: (1) incomplete hydrogen egression, (2) hydrogen trapping in the material after CP sequences, and (3) surface modifications due to charging. The first two points have been widely reported in the literature [40,52]. For the third point, one can see from the SPM images in Figure 6b that the specimen surface did not change in a noticeable manner, and the surface roughness was still in an appropriate range (surface height variation of ~1 nm before test, and ~2 nm after test). Therefore, the irreversible friction due to surface modification can be excluded.

From the pop-in load, the free energy change can be depicted from the model presented in Section 3.4, which describes the HDN procedure. Based on thermodynamics, the critical energy barrier ΔG* (the local maxima of the free energy curves in Figure 10) has to be overcome in order to make the formation of a dislocation loop energetically stable. According to Rice and Beltz [59], the available energy at room temperature for dislocation nucleation is roughly 0.77 eV (calculated by 30*kT*, where *k* is Boltzmann constant and *T* is temperature), which is drawn as a reference line in Figure 10. It can be seen that the free energy curves from the hydrogen-free air condition and the AP and CP1 conditions are below the 0.77 eV threshold, which means that spontaneous dislocation nucleation is thermodynamically possible. Hydrogen charging at CP2 and CP3 clearly made the local maxima of the free energy curves higher than 0.77 eV, and therefore spontaneous HDN under these conditions is energetically unfavorable, and an additional energy source is needed. Comparing the testing conditions, hydrogen charging by cathodic polarization was the only parameter changed between the sequences. Therefore, it is reasonable to conclude that the dissolved hydrogen provided the energy needed for HDN. According to the defactant (defect acting agent) theory [60], hydrogen can act as a defactant that reduces the formation energy of defects such as dislocations. Therefore, the dissolution of hydrogen in the specimen made the nucleation of dislocations easier than the hydrogen-free case, and thus the pop-in load decreased. This effect is also more pronounced with a more negative cathodic potential, i.e., a larger amount of hydrogen generated and ultimately absorbed in the material. CP1 showed a ΔG* at the same level as the AP since in both cases a passive film was present on the surface of the ASS due to the very little hydrogen evolution at CP1. Indeed, hydrogen absorption in the metal is a stepwise process that depends on the diffusion kinetics. Hydrogen evolution reaction proceeds through three reaction steps, (1) Volmer (adsorption), (2) Heyrovsky (electrochemical desorption), and (3) Tafel (chemical desorption), where the latter is typically negligible in an electrochemical process. The hydrogen adsorption and absorption processes cannot be separated, i.e., once hydrogen has been adsorbed on the metal surface, it will absorb into the metal following the Fick’s law [61]. Therefore, a critical amount of hydrogen (driven by the electrode potential applied) is needed in order to trigger significant degradation in the material properties. However, this critical amount is difficult to precisely measure in the material in-situ and in-real-time due to technical limitations. However, using the polarization curve (the current density at a given electrode potential) and assuming that the only cathodic reaction is hydrogen evolution, one could estimate the amount of hydrogen produced on the surface of the stainless steel. The amount of hydrogen absorbed in the material will ultimately depend on the diffusion kinetics and the cathodic potential applied.

### 4.2. Hydrogen Influence on Mechanical Properties

In the current study, a hydrogen-induced softening was observed at the cathodic potential (CP1) that was right at the redox potential for hydrogen (none or very little hydrogen evolution). A weaker softening effect has happened at the other two cathodic potentials (CP2 and CP3) instead of the expected hydrogen-induced hardening effect that is typically observed in conventional ECNI tests at cathodic potentials well below the redox potential for hydrogen evolution [35]. Worth noticing is the short charging periods (only 10 to 30 min per potential sequence) used in this work (Table 2) and the very small volume of electrolyte used. In addition, the electrolyte used was an aqueous electrolyte with very low viscosity, which promotes a fast track for hydrogen bubbles to escape the solution. This can possibly result in very different hydrogen concentration and diffusion in the material compared to other works and the HELP-related softening mechanisms [62]. Though the lattice friction has been enhanced by the introduction of hydrogen, this was modeled only until the onset of plastic deformation, where the massive dislocation motion and the associated dislocation–hydrogen interaction during motion was less pronounced. The hardness measured by ECNI was based on the projected area of the final imprint and the applied load. Since the eventual appearance of the imprint was determined from all four stages of the ECNI (see Figure 3), the elastoplastic loading regime coupled with massive dislocation motion and dislocation–hydrogen interaction could have given a strong contribution to the final hardness.

The hydrogen-induced softening effect observed by uniaxial tensile testing in similar austenitic steels has been reported in literature [63,64]. This can possibly be explained by the HELP mechanism. In 2001, Robertson [65] published the experimental proof for the HELP mechanism that upon in-situ hydrogen charging, the spacing between dislocations became visibly smaller in austenitic steels. This was attributed to the hydrogen-enhanced dislocation mobility. In later years, the conclusion was also applied to other materials such as Ni alloys [66]. It has also been stated that this hydrogen-enhanced mobility of different types of dislocations is working as long as the hydrogen atmosphere can move with the dislocations [66]. The physical reason behind this proposal has been concluded as the reduction effect of hydrogen on the short-range energy barrier of the thermally activated dislocation motion [67,68]. Considering the relatively low amount of hydrogen expected in the current study (specially at CP1), it is not surprising to assume that the environmental conditions of the results obtained in this work fit the conditions of the HELP mechanism, and thus the decrease in hardness should be the expected result.

**Figure 11:**
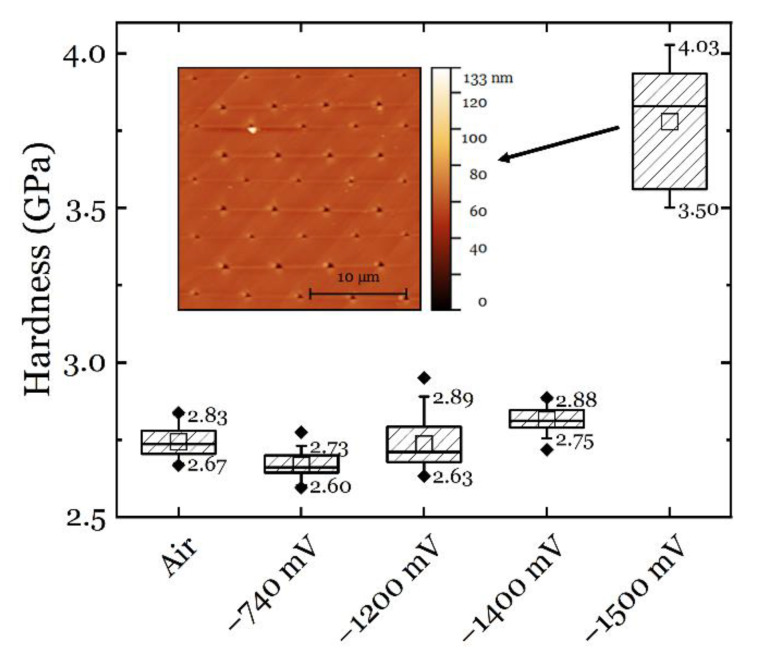
Hardness measured at relatively higher CPs. The hydrogen-free and low CP cases are presented for reference. Note that all measurements were conducted in the same grain and thus the orientation influence can be eliminated.

Indeed, when the hydrogen concentration increases, the material gradually hardens (Figure 7a). As reported in literature, multiple hydrogen-metal interaction mechanisms can be active simultaneously in the material depending on several different parameters, among which the hydrogen concentration is a critical one [12]. As the hydrogen concentration becomes higher than the critical value, the HELP mechanism becomes less dominant, and thus the softening induced by the enhanced dislocation mobility is replaced by other mechanisms. To prove this hypothesis, tests at more negative CPs were performed, and the results of the hardness measurements are shown in Figure 11. It can be seen that when the CP further increased to −1200 mV and −1400 mV, the hardness increased systematically. When the cathodic potential of −1500 mV was reached, the hardness showed an abrupt increase. This increase in hardness was followed by the formation of slip lines and/or martensite formation on the surface, as can be seen from the embedded SPM image in Figure 11. Considering the friction analysis in Section 3.3 and Figure 9, the importance of friction is becoming more and more important with a more negative CP. Therefore, it can be speculated that the friction plays a major role in the hardening behavior at higher hydrogen concentrations, i.e., when only reduction reaction 2 dominates the cathodic charging.

### 4.3. Permanent Change in Material

As mentioned in Section 4.1, the material properties did not fully recover to the level in air condition after the application of AP at +100 mV, and the surface modification was excluded from the possible reasons based on the result in Figure 6b. However, if the CP was further increased to −1500 mV, a clear topographic change could be observed in the material (Figure 11). A similar phenomenon has also been observed in austenitic high-entropy alloys, and the reason for this was the hydrogen-induced internal stress leading HISS (hydrogen-induced surface steps) [69] and/or hydrogen-induced martensitic transformation [70]. The hydrogen-induced surface modifications, either in the form of surface steps or martensitic transformation, can cause irreversible changes in the mechanical performance of the material. Such a permanent change did not happen in CP1~3 and AP charging conditions due to the lack of enough hydrogen and/or the short polarization times. The observed differences in the pop-in behavior and the friction and HDN analyses are therefore the most plausible reason for the intrinsic modification to the material.

On the other hand, the possible martensitic transformation found when the CP was further increased to −1500 mV (Figure 11) shows that the alloy has enough carbon solutes (Table 1) to trigger a permanent transformation in the material. As mentioned in Section 4.1, hydrogen could have generated a Cottrell-like atmosphere that hinders dislocation motion [57]. It may thus be speculated that hydrogen changes the Cottrell atmospheres created by larger solute atoms (carbon or nitrogen, Table 1) by interacting with them. For example, Abraham and Altstetter studied the effect of cathodically charged hydrogen on the yield strength of a 310 s ASS and found an increase in the yield strength with increasing hydrogen content [19]. Since the yielding (discontinuous yielding) can be correlated with the dislocation interactions with the Cottrell atmospheres formed by carbon and nitrogen solutes, it is naturally reasonable to consider the effect of hydrogen on such interactions. This effect was also confirmed by testing pre-deformed specimens, where the interstitial atoms were redistributed due to the plastic deformation [19]. Zielinski et al. proposed that Cottrell atmospheres composed of hydrogen atoms may decrease the dislocation mobility in iron, and thus a change in the internal friction can be observed between the hydrogen-free and the hydrogen-charged iron [71]. The nature of the Cottrell atmosphere (whether created by hydrogen atoms or carbon/nitrogen solutes) needs more advanced techniques to be investigated, but all the discussion supports the conclusion that hydrogen is increasing the internal friction in the material.

## 5. Conclusions

The current work presents a study on the effect of hydrogen in a 316L ASS using in-situ electrochemical nanoindentation (ECNI) techniques. By in-situ polarizing the specimen with different cathodic and anodic potentials, the influence on the nanomechanical properties induced by hydrogen ingression and egression has been examined. The following conclusions have been drawn:

- Cathodic hydrogen charging increases the lattice friction both in the amount and the proportion during the onset of plastic deformation, and the lattice friction recovers towards the hydrogen-free case after anodic discharging.
- The cathodically charged hydrogen promotes the homogeneous dislocation nucleation during nanoindentation, which can be explained by the framework of the defactant theory.
- The softening effect of hydrogen can be observed at cathodic potentials up to the redox potential for hydrogen evolution, which can be explained by the HELP mechanism.
- When charging at higher cathodic potentials (above the redox potential for hydrogen evolution), hydrogen-induced surface steps can be observed, which contribute permanent changes (hardening) to the material.

## Figures and Tables

**Table 1:** Chemical composition of the studied steel.

Element	C	Si	Mn	S	P	Cr	Ni	Mo	N
wt.%	0.015	0.38	1.25	0.0005	0.027	16.39	10.16	2.09	0.04

**Table 2:** Sequence of the testing conditions.

Steps	Testing Condition	Potential	Charging Time	ECNI Time
1	Air	-	0	24 min
2	CP1	−740 mV	30 min	16 min
3	CP2	−808 mV	10 min	23 min
4	CP3	−884 mV	15 min	20 min
5	AP	+100 mV	2 h	13 min

**Table 3:** The constants used in the current study.

Parameter	Symbol	Value	Reference
shear modulus	*µ*	80 GPa	[41]
tip radius	*R*	1875 nm	(extracted from LD curves)
Poisson’s ratio	*ν*	0.3	[41]
Burgers vector	*b*	0.254 nm	[41]
stacking fault energy	*γ*	22.83 mJ/m^2^	[51]

## Data Availability

Data are available on request to the corresponding author.
